# Phospholipids and Alzheimer’s Disease: Alterations, Mechanisms and Potential Biomarkers

**DOI:** 10.3390/ijms14011310

**Published:** 2013-01-10

**Authors:** Marko Kosicek, Silva Hecimovic

**Affiliations:** Division of Molecular Medicine, Rudjer Boskovic Institute, Bijenicka 54, 10 000 Zagreb, Croatia; E-Mail: marko.kosicek@irb.hr

**Keywords:** phospholipid, Alzheimer’s disease, biomarker

## Abstract

Brain is one of the richest organs in lipid content. Phospholipids (glycerophospholipids and sphingolipids) are important building blocks of cell membranes, which provide an optimal environment for protein interactions, trafficking and function. Because of that, alterations in their cellular levels could lead to different pathogenic processes in the brain, such as in Alzheimer’s disease (AD), the most common type of dementia among older populations. There is increasing evidence that phospholipid changes occur during pathogenic processes in AD. It is known that lipids are tightly connected with metabolism of the Amyloid Precursor Protein (APP), which produces Amyloid-beta peptide (Aβ), the main component of senile plaques, which represent the main pathological hallmark of AD. However, the mechanism(s) of the lipid-effect on Aβ metabolism and AD pathogenesis is still not completely understood. This review summarizes the current knowledge on phospholipid changes occurring during normal aging and discusses phospholipid changes in the human brain associated with different stages of AD, as well changes in the cerebrospinal fluid and blood/plasma, which are interesting potential biomarkers for AD diagnosis and disease monitoring. At the end, we have discussed future perspectives of phospholipid changes as potential biomarkers and as targets for development of novel treatment strategies against AD.

## 1. Introduction

Phospholipids are structurally and biologically important molecules, which form cellular membranes and are involved in the behavior of membrane proteins, receptors, enzymes and ion channels intracellularly or at the cell surface. Since the brain is one of the richest organs in lipid content, changes in the brain phospholipid levels could lead to different pathogenic processes. Different regions of the brain differ in phospholipid composition. Development of new and sensitive techniques, such as mass spectrometry imaging, enabled more precise determination of regional phospholipid distribution and provided a powerful tool for clarifying the role of phospholipids in the brain [1]. To differentiate between pathogenic and normal aging process, it is imperative to elucidate what changes in brain phospholipid levels occur during aging. Concentrations of most lipids in the human brain decrease after the age of 50. Phosphatidylinositol (PI), phosphatidylethanolamine (PE) and phosphatidylcholine (PC) brain levels decrease very slowly with age, with less than 10% loss in the period between age 40 and age 100 [2]. In another study, 10%–20% loss of phospholipids in different brain regions was observed only in individuals between age 89 and 92 compared to 33- to 36- year-old individuals, while phospholipid composition in various brain regions stayed unchanged during aging [3]. Phospholipid reduction starts slowly after age 20 and, after age 80, becomes more pronounced, with no significant difference in lipid profile between male and female brains [4,5]. In contrast, ethanolamine plasmalogen (PPE) brain levels decrease 18% (till the age of 70) and 29% (till the age of 100), and sphingomyelin brain levels decrease 12% and 20% in the same time period, respectively [6]. Changes in other lipid classes during aging and the role of lipids in the aging brain were recently extensively reviewed [7].

## 2. Phospholipid Changes in Alzheimer’s Disease

### 2.1. Alzheimer’s Disease

Alzheimer’s disease (AD) is the most common neurodegenerative disorder among older populations. The major risk factor for AD is age, although the exact cause of the disease and pathogenic mechanism of degenerative processes in the brain are still not known. AD is usually developed after age of 65 with the incidence of 67 cases per 1000 in populations older than 65. The risk of disease doubles with every five years of age, with higher rates of incidence for women (men to women rate ratio around 0.5) [8]. Systematic review of epidemiological studies in 2005 estimated the number of AD patients worldwide at 24 million people. Projection says that the number of affected individuals will double every 20 years to 42 million by 2020 and 81 million by 2040. Sixty percent of patients live in developing countries, and the number will rise to 71% by 2040 [9].

The neuropathological characteristics of Alzheimer’s disease are amyloid plaques (aggregates of amyloid-β peptides) and neurofibrillary tangles (formed by accumulation of hyperphosphorylated tau protein), which firstly affect the medial temporal and parietal lobe and part of the frontal cortex of the brain [10]. Amyloid-β (Aβ) peptide is produced by proteolytic processing of the Amyloid Precursor Protein (APP). In the first step, APP is cleaved by either α- or β-secretase producing soluble sAPPα or sAPPβ fragments and membrane-bound *C*-terminal fragments, CTFα or CTFβ, respectively. Both CTFs could be further cleaved by γ-secretase liberating p3 peptide (if the substrate is CTFα) or Aβ (if the substrate is CTFβ) [11]. The availability of APP to α-secretase (non-amyloidogenic pathway) or β-secretase (amyloidogenic pathway) determines how much of the pathogenic Aβ peptide will be produced. Since these two secretases/pathways are likely to be spatially separated within the cell, it is possible that alterations of APP trafficking, caused by lipid changes, may be the primary cause of the disease process.

Certain diagnosis of AD can be made only *post-mortem*. However, today in specialized clinics, using a combination of tools, AD can be diagnosed with more than 95% accuracy. These tools include taking a disease history from patients and their families and assessing cognitive function by neuropsychological tests—the criteria of the National Institute of Neurological and Communicative Diseases and Stroke and the Alzheimer’s disease and Related Disorders Association (NINCDS-ADRDA)—in combination with neuroimaging (CT, MRI and PET) to exclude other causes of dementia [12]. In addition, measurement of cerebrospinal fluid (CSF) levels of Aβ42, tau and phosphorylated tau can help in differential diagnosis of AD and can be useful for predicting AD in individuals with mild cognitive impairment (MCI) [13].

### 2.2. Phospholipid Changes in the Brain of Individuals with Alzheimer’s Disease

The overview of the published work on phospholipid brain levels in AD is shown in Table 1. Publications from late 1980 and 1990 suggested that decreased brain phospholipid levels and alterations in brain phospholipid metabolism could be connected with AD. Decreased PI levels [14,15] and PE levels [14,16,17], especially decrease of PPE relative to PE [18,19], were found in *post-mortem* brain samples from individuals with AD compared to controls. In parallel, a decreased of PC [16,19] or unchanged PC levels [14,17] have been reported. In addition, 73% reduction in choline plasmalogen was observed in frontal cortex from individuals with AD compared to controls [20]. All these studies used traditional analytical techniques for phospholipid profiling, which require larger brain samples that could potentially lead to cross contamination between different brain regions and gray and white matter. Another analytical approach, ^31^P NMR phospholipid profiling, supported those findings and revealed a mild reduction in PI, PE and PC levels in the combined brain regions’ lipid extracts from individuals with AD compared to non-demented controls [21]. Development of mass spectrometry provided a great tool for lipidomics, enabling simultaneous analysis of more phospholipid classes using a small sample size. Han and coworkers developed an electrospray ionization mass spectrometry (ESI-MS) platform for sensitive phospholipid profiling and showed different changes of PPE levels during AD development between gray and white matter. Deficiency of PPE in the white matter was already around 40% in an early stage of AD and was constant during disease progression, while deficiency of PPE in the gray matter was gradually increased from 10% to 30% during disease progression [22]. Interestingly, in an early stage of AD, brain levels of other phospholipid classes (PC, PI and PE) in both white and gray matter were unchanged [23].

Beside glycerophospholipid changes, alterations in the brain sphingophospholipid levels also occur during AD. *Post-mortem* brain analysis gave contradictory results about sphingomyelin (SM) levels in AD brain, depending on the brain tissue analyzed (e.g., whole brain extract or specific brain region; separation or no separation of white and gray matter) and on the used analytical approach. Decreased SM levels were observed in soluble fractions, but unchanged in membrane fractions from AD brains compared to control brains using enzymatic assay [24]. Significantly decreased levels of SM were reported in the middle frontal gyrus of AD patients compared with controls, but were not decreased in the cerebellum [25]. In contrast, Bandaru and coworkers found significant increase of SM in middle frontal gyrus gray matter, while in middle frontal gyrus white matter, there were no differences in SM levels using ESI-MS [26]. ^31^P NMR approach found an increase in SM levels in combined brain regions from AD compared to controls, but not in all brain regions. Interestingly, there was statistically significant increase of SM in the cerebellum, but not in the superior/middle frontal gyrus, which showed a slight, but not statistically significant, increase in SM levels [21]. In an early stage of AD, SM levels in gray and white matter were unchanged in both frontal cortex and cerebellum [23]. Even though the results on SM levels in AD brains are confusing, there is a strong evidence that ceramide, the main precursor in sphingolipid metabolism, is increased in AD brains [23–25]. The most affected sphingolipid in AD brain is sulfatide, which is depleted up to 93% in gray matter and up to 58% in white matter [23]. Sphingolipid metabolism changes in AD have been thoroughly reviewed elsewhere [27–29]. To explore whether these phospholipid changes could be used for AD diagnosis or for monitoring the disease progression/treatment, it is important to determine if their changes could be observed in a sample more appropriate for diagnostic purposes, such as CSF or blood.

### 2.3. Phospholipid Changes in the CSF and Blood of Individuals with Alzheimer’s Disease

CSF is the most informative fluid source for neurodegenerative disease diagnosis/research, because of its constant physical contact with brain. Despite the fact that many phospholipid alterations in the AD brain have been reported, only a few studies attempted to monitor those changes in the CSF (Table 2). Unchanged PC levels, but decreased lysoPC/PC ratio, have been reported in the CSF from individuals with AD compared to individuals with memory complaints, but without dementia [30], together with elevation in different PC metabolites, suggesting that AD is accompanied with increased PC hydrolysis [31]. Sphingolipid alterations found in AD brain were also observed in the CSF. Increased levels of ceramides have been found in the CSF from individuals with AD compared to age-matched individuals with amyotrophic lateral sclerosis (ALS) and other neurological controls [32]. Han and coworkers found approximately 40% reduction of sulfatides in the CSF in an early stage of AD and unchanged levels of PI. The authors suggested that CSF sulfatide/PI ratio could be a sensitive and specific biomarker for early AD diagnosis [33]. Recently, we developed a high-throughput-amenable and sensitive HPLC–ESI/MS method for CSF phospholipid screening [34] and examined phospholipid levels in the CSF from AD patients representing different stages of the disease, including prodromal AD. We observed a statistically significant increase (around 50%) in SM levels between prodromal AD group and cognitively normal group, while PI, PE and PC levels were unchanged in all examined groups [35].

Although CSF is the most informative sample for monitoring brain pathological processes, blood/plasma is much easier and less invasive to collect and, thus, is more suitable for routine diagnosis and/or disease monitoring. Due to blood-brain barrier and huge influence of diet and other body processes outside the brain on blood lipid levels, it is difficult to make a direct link between blood phospholipid levels and neurodegenerative processes. However, Mielke and coworkers reported a connection between lower levels of SM and ceramides and memory impairment [36]. In addition, they found significantly lower ceramide levels in plasma from individuals with mild cognitive impairment, while no difference was observed in plasma ceramide levels between AD and non-demented controls [37]. Potential usage of peripheral sphingolipid as biomarkers of AD was recently reviewed elsewhere [38].

## 3. Lipids as Biomarkers for Alzheimer’s Disease Diagnosis and as Targets for Potential Novel Treatment Strategies

As we pointed out earlier, currently, the diagnosis of AD is based on the combination of different tests/methods for the exclusion of other causes of dementia. Neurological tests, which are still the gold standard for the diagnosis of AD, are mostly accurate in identifying individuals with already developed dementia. The biggest challenge for clinicians and for application of novel therapies against AD is to accurately recognize prodromal AD patients and/or individuals with mild cognitive impairment (MCI) who will develop AD. Studies suggest that CSF biomarkers p-tau, total tau and Aβ42 are useful for AD diagnosis and for identifying individuals with prodromal AD in MCI cases [13]. The multicenter study found that these CSF biomarkers identify incipient AD with good accuracy, but with less accuracy compared to single-center studies. Additional effort is needed for standardization of analytical techniques and clinical procedures to avoid variability between different centers [39]. Although so far reported CSF/blood phospholipid changes are not specific and sensitive enough to be a diagnostic biomarker, phospholipid alterations could lead to membrane instability and synaptic loss and, in that way, contribute to AD pathology [18,20]. However, sphingolipid alterations are promising candidates as AD biomarkers.

### 3.1. Sulfatides

The most promising sphingolipid candidate for early AD diagnosis is reduction of CSF sulfatide levels [33]. Sulfatide brain levels were not reduced in dementia with Lewy bodies and were elevated in different brain regions of individuals with Parkinson’s disease [40]. CSF sulfatide levels in individuals with vascular dementia were elevated compared to controls and individuals with AD [41], and low CSF sulfatide levels could predict progression of white matter lesions [42]. However, specificity and sensitivity of sulfatide as a potential biomarker of AD has yet to be determined.

Mechanistic studies suggest that apolipoprotein E (apoE) plays an important role in the regulation of sulfatide levels in the brain [43]. ApoE is a primer apolipoprotein in the brain, which mediates the transport of cholesterol, triacylglycerides, phospholipids and sulfatides in the brain. In humans, ApoE is present in three isoforms. The most common isoform is apoE3 (with Cys 112 and Arg 158), following by apoE2 (with Cys 112 and Cys 158) and apoE4 (with Arg 112 and Arg 158). So far, apoE4 has been proven to be a genetic risk factor for AD [44]. ApoE can modulate Aβ metabolism and accumulation in apoE isoform-dependent manner—Aβ accumulation rises in the order apoE2, apoE3 and apoE4. Although the mechanism is not completely understood, it seems that apoE can bind Aβ and prevent formation of toxic Aβ oligomers and fibrils [45]. Furthermore, neurotoxic microglial activation also depends on apoE phenotype and rises in the order apoE2, apoE3 and apoE4, suggesting that apoE can modulate AD by multiple apoE isoform-specific mechanisms [46]. ApoE regulates brain sulfatide levels by transporting sulfatide from cells to the CSF or by endocytic recycling of sulfatide-containing apoE particles. In addition, cognitively normal individuals with one or two apoE4 alleles have more sulfatides in the CSF compared to apoE3 homozygous, suggesting that apoE may be involved in the sulfatide loss in AD [47]. The exact connection between sulfatide deficiency and AD is still not clear, but CSF sulfatide levels alone or in combination with other biomarkers could contribute to early AD diagnosis and/or monitoring of therapeutic treatments [48].

### 3.2. Ceramide

Ceramide is the major precursor in sphingolipid metabolism and a powerful second messenger that regulates growth inhibition, apoptosis and stress response. Together with other bioactive sphingolipids, it is important for neuronal signaling and function [49,50]. Elevation of ceramide levels in AD reaches the highest point in MCI cases. That is probably the result of sulfatide degradation, which occurs in an early stage of AD since the molecular species profile of ceramides is similar to the molecular species profile of sulfatides in individuals with MCI [23]. The study, which examined changes in the expression of genes coding ceramide metabolism enzymes, found both upregulation of genes involved in *de novo* synthesis of ceramide and downregulation of genes involved in glycosphingolipid synthesis. Those changes were visible in an early stage of AD, confirming that observed changes of sulfatide and ceramide levels in AD brain are connected with disturbed sphingolipid metabolism [51]. *In vitro* studies suggest that Aβ activates sphingomyelin hydrolysis and causes ceramide accumulation [25,52]. Sphingomyelin can be hydrolyzed by acid, alkaline or neutral sphingomyelinases named after the pH at which they are the most active. In AD, brain increased acid sphingomyelinase activity has been reported [24], while *in vitro* and animal studies suggest involvement of both acid and neutral sphingomyelinases in ceramide accumulation due to Aβ stimulation. Ceramide can also influence Aβ production by stabilizing β-secretase (BACE1) and promoting amyloidogenic processing of APP [53,54]. Although there is no clear mechanism, which could explain the ceramide role in Aβ cytotoxicity, several mechanisms have been proposed [55]. Because of its connection with pathobiological processes and changes in CSF and plasma levels in an early stage of AD, ceramide could potentially be a candidate as an AD biomarker.

### 3.3. Lipid Rafts

Since all enzymes involved in APP processing (α-, β- and γ-secretase) are transmembrane proteins, as well as APP and its *C*-terminal fragments (αCTF and βCTF), it is logical to assume that lipid within the membrane bilayer may play an important role in APP processing and Aβ production. Lipid changes in AD (especially in cholesterol and sphingolipid metabolism) suggest that lipid rafts could be involved in AD pathogenesis. By definition lipid rafts are 10–200 nm small, heterogeneous, highly dynamic membrane domains enriched in sterol and sphingolipids [56]. Lipid rafts have many alternative names, mostly based on the method of their isolation (e.g., detergent resistant membranes DRM). There are different methods for lipid raft isolation and different theoretical models about their structure and formation [57]. Numerous studies suggested that amyloidogenic processing of APP occurs in lipid rafts, since APP, β-secretase (BACE1) and γ-secretase are localized in lipid rafts [58–61]. In addition, we reported that increased levels of cholesterol and sphingolipids in the Niemann-Pick type C (NPC) disease cellular model lead to increased localization of APP in lipid rafts, which supports the link between NPC disease and AD [62]. Under normal conditions, only a small portion of BACE1 is localized in lipid rafts. Targeting of BACE1 to lipid rafts by adding an glycosylphosphatidylinositol (GPI) anchor at the place of transmembrane and *C*-terminal domain increased the production of sAPPβ and Aβ [63]. In contrast, α-secretase (ADAM10) is under normal conditions exclusively present in non-raft fractions. Replacing the transmembrane and cytosolic domain of ADAM10 with GPI anchor caused retargeting of ADAM10 to lipid rafts and reduced amyloidogenic APP processing [63]. Overall, these results imply that regulation of lipid rafts protein targeting could be a good approach for controlling APP amyloidogenic processing. However, another signal for BACE1 targeting to lipid rafts is S-palmitoylation of Cys 474, 478, 482 and 485. Mutations of these Cys residues to Ala relocated BACE1 out of lipid rafts, but without affecting APP amyloidogenic processing [64]. The same approach revealed that *S*-palmitoylation of γ-secretase complex subunits nicastrin and APH1 is important for their stability and raft localization, but not for γ-secretase processing of APP [65]. Another interesting approach was the synthesis of the membrane-anchored version of BACE1 inhibitor, which was targeted to endosomes and lipid rafts, where its local concentration was increased. In that way, this inhibitor was more potent and focused on active BACE1 [66]. Although the role of lipids rafts in AD pathogenesis is still controversial (as lipid rafts are controversial *per se*), it is evident that specific membrane platforms are involved in APP, BACE1 and γ-secretase colocalization, APP processing and formation of the pathogenic Aβ peptide.

## 4. Conclusions

Although the focus of this review is on phospholipids, it is important to add that cholesterol and fatty acid changes were also observed in AD brains and connected with AD development. These findings were extensively reviewed elsewhere [67–69]. Phospholipids provide an optimal membrane environment for protein interactions, trafficking and function. There is increasing evidence that phospholipid changes occur during pathogenic processes in Alzheimer’s disease. Although so far reported CSF/blood phospholipid changes are not specific and sensitive enough to be a diagnostic biomarker, a combination of different sphingolipid CSF and/or blood levels could potentially contribute to more precise AD diagnosis in a very early stage. However, further research is required in order to clearly state that any of those lipids could be used as a biomarker.

## Figures and Tables

**Table 1:** Phospholipid changes in the brain of individuals with Alzheimer’s disease.

Lipid class	Change/Normalization	Sample size/Examined brain regions/Analytical method	Reference
PI	decreased/wet weight	9 AD and 9 controls/HPG, SMTG, IPL and cerebellum/TLC	[14]
PI	decreased/wet weight	17 AD and 18 controls/anterior temporal cortex/TLC	[15]
PI	decreased/relative	45 AD and 11 controls/SMFG, STG, IPL, occipital cortex and cerebellum/^31^P NMR	[21]
PE	decreased/wet weight	9 AD and 9 controls/HPG, SMTG, IPL and cerebellum/TLC	[14]
PE	decreased/DNA	10 AD and 10 controls/frontal, primary auditory and parietal cortex/photometrical phosphorus determination	[16]
PPE	decreased/relative	9 AD and 9 controls/middle-temporal cortex/HPLC and TLC	[18]
PPE	decreased/phosphate	15 AD and 13 controls/frontal cortex, hippocampus and white matter/HPLC and GC	[19]
PPE	decreased/relative	45 AD and 11 controls/SMFG, STG, IPL, occipital cortex and cerebellum/^31^P NMR	[21]
PPE	decreased/protein	6 CDR = 0; 6 CDR = 0.5; 6 CDR = 1; 6 CDR = 2; 6 CDR = 5/white and gray matter from SFG, STG, IPL and cerebellum/ESI-MS	[22]
PC	unchanged/wet weight	9 AD and 9 controls/HPG, SMTG, IPL and cerebellum/TLC	[14]
PC	decreased/DNA	10 AD and 10 controls/frontal, primary auditory and parietal cortex/HPLC–fluorimetric detection	[16]
PC	unchanged/wet weight	6 AD and 4 controls/gray matter from frontal cortex, parietal and temporal region/HPLC	[17]
PC	decreased/phosphate	15 AD and 13 controls/frontal cortex, hippocampus and white matter/HPLC and GC	[19]
SM	decreased/protein	9 AD and 6 controls/gray matter from frontotemporal area/enzymatic assay–HPLC	[24]
SM	decreased/relative	7 AD and 7 controls/MFG, SFG and cerebellum/ESI-MS	[25]
SM	increased/relative	30 AD and 26 controls/MFG, MTG and cerebellum/ESI-MS	[26]
SM	increased/relative	45 AD and 11 controls/SMFG, STG, IPL, occipital cortex and cerebellum/^31^P NMR	[21]
ceramide	increased/protein	6 CDR = 0; 6 CDR = 0.5; 6 CDR = 1; 6 CDR = 2; 6 CDR = 5/white and gray matter from MFG, STG, IPL and cerebellum/ESI-MS	[22]
ceramide	increased/protein	9 AD and 6 controls/gray matter from frontotemporal area/enzymatic assay–HPLC	[24]
ceramide	increased/relative	7 AD and 7 controls/MFG, SFG and cerebellum/ESI-MS	[25]
sulfatide	decreased/protein	6 CDR = 0; 6 CDR = 0.5; 6 CDR = 1; 6 CDR = 2; 6 CDR = 5/white and gray matter from MFG, STG, IPL and cerebellum/ESI-MS	[23]

**Table 2:** Phospholipid changes in the cerebrospinal fluid (CSF) of individuals with Alzheimer’s disease.

Lipid class	Change	Sample size/Sample collection	Reference
PC	decreased lysoPC/PC	30 AD and 31 controls/*post mortem*	[30]
PC	increased PC metabolites	12 AD and 30 controls/lumbar puncture	[31]
SM	increase in prodromal AD	21 AD and 16 controls/lumbar puncture	[35]
ceramide	increase	16 AD and 14 controls/lumbar puncture	[32]
sulfatide	decrease	19 CDR = 0; 20 CDR = 0.5/lumbar puncture	[33]

## Abbreviations

Aβ: Amyloid beta peptide
AD: Alzheimer’s disease
Apo E: apolipoprotein E
APP: Amyloid Precursor Protein
CDR: clinical dementia rating
CSF: cerebrospinal fluid
CTF: Amyloid Precursor Protein *C*-terminal fragments
ESI-MS: electrospray mass spectrometry
GC: gas chromatography
HPG: subiculum of the hippocampus and parahippocampal gyrus
HPLC: high-performance liquid chromatography
IPL: inferior parietal lobe
MFG: middle frontal gyrus
MTG: middle temporal gyrus
PC: phosphatidylcholine
PE: phosphatidylethanolamine
PI: phosphatidylinositol
PPE: ethanolamine plasmalogen
sAPP: soluble Amyloid Precursor Protein *N*-terminal fragments α or β
SM: sphingomyelin
SMFG: superior-middle frontal gyrus
SMTG: superior-middle temporal gyrus
SFG: superior frontal gyrus
STG: superior temporal gyrus
TLC: thin layer chromatography
